
JOURNAL INFORMATION
==============================
NLM Title Abbreviation: Wirtschaftsdienst
Journal ID: Wirtschaftsdienst
NLM Journal ID: 101086612

Wirtschaftsdienst (Hamburg, Germany : 1949)

ISSN: 0043-6275

ARTICLE INFORMATION
==============================
PMCID: PMC7896175
PMID: 33642645
DOI: 10.1007/s10273-021-2849-x
Article ID: 2849
Article version: 1
Subjects: Zeitgespräch

Reform der EU-Fiskalregeln nach Corona wichtiger denn je

Truger Achim achim.truger@uni-due.de

grid.5718.b 0000 0001 2187 5445 Institut für Sozioökonomie, Universität Duisburg-Essen, Lotharstr. 65, 47057 Duisburg, Deutschland
Electronic publication date: 2021 Feb 20
Print publication date: 2021
Volume: 101
Issue: 2
First page: 94
Last page: 98
Copyright: © Der/die Autor:in(nen) 2021
License: Open Access: Dieser Artikel wird unter der Creative Commons Namensnennung 4.0 International Lizenz veröffentlicht (http://creativecommons.org/licenses/by/4.0/deed.de).
Open Access wird durch die ZBW — Leibniz-Informationszentrum Wirtschaft gefördert.
License URL: https://creativecommons.org/licenses/by/4.0/

issue-copyright-statement: © The Author(s) 2021

==============================
Prof. Dr. Achim Truger ist Professor für Sozioökonomie mit dem Schwerpunkt Staatstätigkeit und Staatsfinanzen an der Universität Duisburg-Essen und Mitglied des Sachverständigenrates zur Begutachtung der gesamtwirtschaftlichen Entwicklung.

Europa befindet sich noch mitten in der durch die Corona-Pandemie verursachten schweren Wirtschaftskrise. In dieser Situation stellt der Europäische Aufbauplan insbesondere mit der Aufbau- und Resilienzfazilität eine wesentliche Unterstützung für die besonders von der Krise getroffenen Mitgliedstaaten dar. Der Erfolg des Aufbauplans wird jedoch massiv bedroht, sollten die in der Krise ausgesetzten EU-Fiskalregeln in den kommenden Jahren in unveränderter Form wiedereingesetzt werden. Die dringend notwendigen expansiven Impulse könnten dann durch eine erneute Austeritätswelle zunichte gemacht werden. Die Reformbedürftigkeit der Fiskalregeln war bereits lange vor der Krise offensichtlich. Nun ist eine Reform, die den Stabilitäts- und

Wachstumspakt flexibler, investitionsorientierter und demokratischer macht, umso wichtiger.

### Schwere Krise mit hohen fiskalischen Belastungen

Der coronabedingte Einbruch des Bruttoinlandsprodukts von -7,5 % in der EU und -7,8 % im Euroraum ist laut Herbstprognose der EU-Kommission (2020a) der stärkste seit dem zweiten Weltkrieg, und trifft ausgerechnet die schon von der Eurokrise erschütterten Staaten Italien, Spanien, Portugal und Griechenland besonders hart. Das reale BIP der genannten vier Staaten wird laut Herbstprognose der EU-Kommission im laufenden Jahr trotz unterstelltem deutlichem Aufschwung unterhalb des Niveaus von vor der globalen Finanz- und Wirtschaftskrise im Jahr 2008 liegen. In Griechenland wird der gesamte Aufschwung seit dem Jahr 2016 wieder zunichte gemacht, und Italien wird nach langer stagnativer Wirtschaftsentwicklung auf das BIP-Niveau von 1999 zurückgeworfen. Aufgrund des Wirtschaftseinbruchs und der notwendigen finanzpolitischen Unterstützungsmaßnahmen hat sich die Lage der öffentlichen Finanzen nach vielen Jahren der Konsolidierung wieder dramatisch verschlechtert, und die Schuldenstandsquoten werden kräftig ansteigen. Anders als in der Eurokrise seit dem Jahr 2010, als der Euroraum durch falsche Politik in eine Austeritätskrise gezwungen wurde, reagierte die EU in der Corona-Krise wirtschaftspolitisch insgesamt richtig, indem umgehend Unterstützungsmaßnahmen eingeleitet wurden (SVR, 2020, 166 ff.).

### Erfolgreiche EU-Krisenreaktion

Die EZB stellte schnell Liquidität bereit und stabilisierte die Anleihemärkte. Die EU setzte den Stabilitäts- und Wachstumspakt aus. Im Mai 2020 wurde ein Sicherheitsnetzpaket im Umfang von 540 Mrd. Euro mit Kreditlinien für alle Mitgliedstaaten verabschiedet. Dabei handelte es sich um drei zusätzliche Unterstützungsprogramme. Das EU-Kurzarbeiterprogramm SURE, eine zusätzliche Kreditlinie beim Europäischen Stabilitätsmechanismus (ESM) sowie ein Kreditprogramm der Europäischen Investitionsbank. Schließlich kam es nach einigem politischen Ringen zur Verabschiedung des Europäischen Aufbauplans, in dessen Aufbau- und Resilienzfazilität auch direkte, nicht rückzahlbare Unterstützungsleistungen an die Mitgliedstaaten in erheblichem Umfang enthalten sind. Finanziert wird der Fonds durch Kredite der EU, die aus dem EU-Haushalt bedient werden, wobei hierfür perspektivisch neue eigene EU-Steuereinnahmen vorgesehen sind.

Nachdem die Reform der fiskalischen Governance in der EU vor der Krise weitgehend zum Erliegen gekommen war (Seikel und Truger, 2019), stellt das beschlossene Wiederaufbauprogramm offensichtlich einen großen politischen Fortschritt dar. Allerdings bleibt abzuwarten, ob und in welchem Umfang es tatsächlich zu eigenen EU-Steuereinnahmen kommen und ob es gelingen wird, dauerhaft ein fiskalisches Stabilisierungsinstrument für die EU oder den Euroraum zu etablieren. Für die nächsten Jahre hat die Aufbau- und Resilienzfazilität aus makroökonomischer Sicht mit 672,5 Mrd. Euro (4,8 % des Vorkrisen-BIPs der EU im Jahr 2019) für 2021 bis 2023, davon allein 312,5 Mrd. Euro (2,2 % des BIP) als nicht rückzahlbare direkte Zuschüsse, jedenfalls eindeutig ein relevantes Volumen. Für die vier besonders betroffenen Krisenländer liegt das Volumen sogar bei 3,7 % (Italien), 4,8 % (Spanien), 6,2 % (Portugal) und 8,9 % des BIP (Griechenland) (EU-Kommission, 2020b). Wenn man davon ausgeht, dass die Mittel größtenteils zusätzlich zur bisherigen Finanzplanung verwendet werden und einen hohen Investitionsanteil aufweisen, dann sollte sich damit sowohl kurzfristig wegen eines hohen Multiplikators als auch langfristig eine sehr erhebliche positive Wachstumswirkung erzielen lassen. So kommen Dullien et al. (2021) unter Berücksichtigung auch der in der Fazilität enthaltenen Darlehen, aber unter Verwendung des NIGEM-Modells mit geringen Multiplikatoren kumuliert bis zum Jahr 2026 zu positiven BIP-Effekten von über 2 % für den Euroraum und sogar 4 % bis 7,5 % für die genannten Krisenländer.

### Gefahr erneuter Austerität nicht gebannt

Die Freude über die in Aussicht stehenden positiven Effekte des Wiederaufbaufonds sollte aber nicht den Blick auf die Gefahren verstellen, die drohen, sollte das fiskalische Regelwerk des Stabilitäts- und Wachstumspakts (SWP) schon bald wieder ohne Änderungen in Kraft gesetzt werden. Wie erläutert hat die Corona-Krise im Euroraum zu einem Rekordeinbruch bei der Wirtschaftsleistung geführt, wobei gerade die ohnehin lange Zeit kriselnden Staaten Italien, Spanien, Portugal und Griechenland besonders stark betroffen sind. Es sollte offensichtlich sein, dass diese Staaten keine erneute strikte Haushaltskonsolidierung oder gar eine neue Austeritätsphase verkraften können.

**Prof. Dr. Achim Truger** ist Professor für Sozioökonomie mit dem Schwerpunkt Staatstätigkeit und Staatsfinanzen an der Universität Duisburg-Essen und Mitglied des Sachverständigenrates zur Begutachtung der gesamtwirtschaftlichen Entwicklung.

Eine baldige Wiedereinsetzung des SWP in unveränderter Form könnte insbesondere die Krisenstaaten schnell wieder zu stark restriktiver Fiskalpolitik zwingen. Für 2022 wird für den Euroraum insgesamt ein Budgetdefizit von 4,7 % des BIP und für die Krisenstaaten zwischen 3,0 % (Portugal) und 8,6 % (Spanien) prognostiziert. Die Prognosen für die strukturellen Budgetdefizite liegen bei 3,7 % für den Euroraum insgesamt und zwischen 1,1 % (Griechenland) und 7,2 % des BIP (Spanien). Die betroffenen Staaten würden also wegen der strukturellen Defizite schnell in Konflikt mit dem präventiven Arm des SWP geraten, sodass sie zügig zum Abbau ihrer strukturellen Defizite gezwungen wären. Zudem würde gegen einige der Staaten auch bereits wieder ein Verfahren wegen eines übermäßigen Defizits eingeleitet. Zusätzlich würden die um 10 bis knapp 30 Prozentpunkte stark angestiegenen Schuldenstandsquoten gegebenenfalls noch zu einem schuldenstandsbezogenen Defizitverfahren führen. Die dadurch ausgelöste fiskalische Restriktion würde die positiven Effekte des Wiederaufbaufonds konterkarieren sowie die konjunkturelle Erholung gefährden und die Wirtschafts- und Beschäftigungsaussichten dramatisch verschlechtern. Die gravierenden ökonomischen, sozialen und politischen Probleme würden den Fortbestand des Euro und höchstwahrscheinlich auch der EU insgesamt gefährden. Dies würde selbstverständlich auch Deutschland und zudem die sogenannten sparsamen Vier (Österreich, die Niederlande, Dänemark und Schweden) in eine schwere ökonomische Krise stürzen – von den unwägbaren politischen Turbulenzen ganz abgesehen.

## Stabilitäts- und Wachstumspakt ohnehin reformbedürftig

Neben der Verabschiedung des EU-Wiederaufbaufonds sind daher Reformen des SWP im gesamteuropäischen wie im deutschen Interesse notwendig.[1] Der Wiederaufbaufonds kann bei entsprechender Ausgestaltung zwar entscheidende Wachstumsimpulse auch in den bislang wirtschaftlich schwächeren Staaten setzen. Jedoch ist er lediglich als einmalige Maßnahme angesichts der außergewöhnlichen Tiefe der Corona-Krise angelegt. Selbst wenn er auf Dauer angelegt wäre, bliebe das fiskalische Regelwerk des SWP dringend reformbedürftig, weil es von Anfang an unter schwerwiegenden Mängeln litt. Die Grenzwerte von drei Prozent des BIP für das Budgetdefizit und von 60 Prozent des BIP für die Schuldenstandsquote sind aus ökonomischer Sicht völlig willkürlich und stehen zudem häufig in faktischem Widerspruch zu anderen offiziellen Zielwerten, etwa denen von „Europe 2020“ oder denen des Verfahrens gegen übermäßige ökonomische Ungleichgewichte. Vor allem aber hat sich die Unzulänglichkeit des SWP während der Eurokrise unter Beweis gestellt. Er erwies sich als prozyklisch und krisenverschärfend und konnte insbesondere den dramatischen Absturz der öffentlichen Investitionen nicht verhindern (Truger, 2020).

1 Die folgenden Abschnitte lehnen sich stark an Truger (2020) und vor allem (2021) an.

Eine Reform des SWP sollte mindestens drei wesentliche Punkte adressieren: Erstens sollte die konjunkturelle Flexibilität deutlich vergrößert werden. Zweitens bedarf es realistischerer Zielvorgaben und einer klaren Investitionsorientierung. Drittens schließlich bedarf es einer stärkeren Wohlstandsorientierung und einer Demokratisierung der Verfahren.

## Stabilitäts- und Wachstumspakt flexibilisieren

Ein wesentlicher Grund für die mangelnde konjunkturelle Flexibilität des aktuellen SWP besteht darin, dass die – prinzipiell sinnvollerweise – zur Konjunkturbereinigung verwendeten Verfahren nur sehr unvollkommen funktionieren. Daher könnte eine diesbezüglich sehr wirkungsvolle Überarbeitung des SWP sogar rein technisch ohne Änderungen des EU-Rechts erfolgen: Denkbar wäre die grundsätzliche Verwendung mittelfristiger Durchschnittswerte für das Potenzialwachstum oder auch eine lediglich mittelfristige Revision der Potenzialschätzungen, z. B. nur alle drei oder fünf Jahre und nicht wie heute dreimal jährlich. Allein dadurch könnte schon verhindert werden, dass Staaten im Abschwung aufgrund einer rein technisch bedingten Abwärtsrevision der mittelfristigen Wachstumserwartungen in krisenverschärfende Kürzungspolitik getrieben werden. Im Aufschwung müsste umgekehrt schneller und kräftiger konsolidiert werden (Truger, 2015a). Eine weitere pragmatische Änderung zur Vergrößerung der fiskalischen Spielräume wäre es, wenn die EU-Kommission ihre auf Jean-Claude Juncker zurückgehende weniger strenge Interpretation des fiskalischen Regelwerks (Europäischer Rat, 2015) noch etwas großzügiger auslegen würde (Seikel und Truger, 2019).

## Realistischere Zielwerte und Investitionsorientierung

Angesichts der nach der Krise zu erwartenden stark gestiegenen Schuldenstände ist es unrealistisch, die betroffenen Mitgliedstaaten den unveränderten Referenzwert von 60 % des BIP anstreben zu lassen. Die hierzu notwendigen Primärüberschüsse (European Fiscal Board, 2020, 85 ff.) würden über einen langen Zeitraum zu sehr restriktiver Fiskalpolitik zwingen. Um dies zu verhindern, sollte der Zielschuldenstand im Rahmen des SWP deutlich nach oben, z. B. auf 90 % oder 100 % des BIP, angepasst werden. Der gegenwärtige Grenzwert von 60 % entbehrte bekanntlich einer ökonomischen Fundierung und entsprach letztlich auch nur dem bei Verabschiedung der Maastricht-Kriterien herrschenden EU-Durchschnittswert. Für eine Anhebung der Schuldengrenze spricht auch, dass sich aufgrund des gegenwärtig und auf absehbare Zeit sehr niedrigen Zinsniveaus die Bedingungen für die Tragfähigkeit der Staatschulden erheblich verbessert haben. Passend zur höheren Schuldengrenze könnten auch die direkt oder indirekt damit korrespondierenden Defizitgrößen im SWP, also die Drei-Prozent-Grenze für ein übermäßiges Haushaltsdefizit und der mittelfristige Zielwert für einen strukturell ausgeglichenen Haushalt, entsprechend angepasst werden.

Mindestens ebenso wichtig wäre eine investitionsorientierte Reform des SWP. Wie während der Eurokrise zu beobachten, werden in Konsolidierungsphasen regelmäßig die öffentlichen Investitionen stark gekürzt, weil sie eine der wenigen kurzfristig disponiblen Ausgabenkategorien darstellen. Makroökonomisch ist dies jedoch äußerst schädlich, da öffentliche Investitionen sowohl kurzfristig besonders hohe Multiplikatoren aufweisen (Gechert, 2015) als auch langfristig besonders wachstumsfördernd sind (Bom und Ligthart, 2014). Daher sollte ein reformierter SWP die öffentlichen Investitionen stärken und in Krisen vor Kürzungen bewahren. Hierfür sollten die Nettoinvestitionen aus den relevanten Defizitgrößen des präventiven und des korrektiven Zweigs des SWP herausgerechnet werden. Damit würde endlich die Goldene Regel der öffentlichen Investitionen verankert (Truger, 2015b). Die Goldene Regel ist in der traditionellen finanzwissenschaftlichen Literatur allgemein akzeptiert. Sie schreibt die Finanzierung von öffentlichen Nettoinvestitionen durch Budgetdefizite vor, wodurch gleichzeitig der Generationengerechtigkeit wie dem Wirtschaftswachstum gedient wäre. Öffentliche Investitionen erhöhen den öffentlichen Kapitalstock und schaffen Wachstum zugunsten zukünftiger Generationen. Deswegen ist es gerechtfertigt, zukünftige Generationen auch zur Finanzierung über den Schuldendienst heranzuziehen. Sollte die Abgrenzung der öffentlichen Investitionen als zu kontrovers und die Berechnung der Abschreibungen als zu schwierig eingeschätzt werden, könnte man auch jeweils vor einer oder mehreren Budgetperiode(n) die für privilegierungswürdig erachteten Bruttoausgaben in klarer Abgrenzung vorgeben und durch eine Obergrenze von z.B. 1,5 % oder 2 % des BIP die Tragfähigkeit sicherstellen.

### Stärkere Wohlstandsorientierung und Demokratisierung

Über die zuvor beschriebenen Reformvorschläge hinaus braucht die Finanzpolitik in Europa eine grundsätzliche Neuorientierung weg von technokratischen Grenzen für Defizite oder Schulden und hin zu einer Orientierung am Ziel eines nachhaltigen und sozialen Wohlstands (Alvarez et al., 2019, 20 ff.). Die Finanzpolitik sollte Teil eines breit angelegten Rahmens für die ökonomische und soziale Governance sein, die sich beispielsweise am „Wohlstand der Völker“ oder „nachhaltigen gesellschaftlichen Fortschritt“ orientiert. Beide Prinzipien sind in den Europäischen Verträgen angesprochen (Feigl, 2017); sie finden sich in der UN-Agenda für Nachhaltige Entwicklung (UN, 2015) und wurden auch von Stiglitz et al. (2018) aufgegriffen.

Um das Ziel nachhaltigen, hochwertigen Wohlstands zu erreichen, bedarf es einer Politikkoordination, die weit über numerische Fiskalziele hinausgeht. Sie könnte auf die Zielsetzungen materieller Wohlstand, Vollbeschäftigung, Lebensqualität sowie ökologische und ökonomische Nachhaltigkeit ausgerichtet sein. Die Mitgliedstaaten sollten dann eine wirtschaftspolitische Strategie verfolgen, die sich an gemeinsam verabschiedeten ökonomischen, sozialen und ökologischen Zielen orientiert (Mersch, 2018). Ein pragmatischer Vorschlag hierzu wäre die Entwicklung eines integrierten Indikatorensystems. Bei Zielverfehlungen sollte mittels koordinierter Politik eingegriffen werden. So könnte z.B. ein „Annual Well-being and Convergence Survey (AWCS)“ erstellt werden, das jedes Jahr zu Beginn des Europäischen Semesters im EU-Parlament, im Europäischen Wirtschafts- und Sozialausschuss (EWSA) und im Makroökonomischen Dialog diskutiert werden müsste, bevor die politischen Prioriäten gesetzt werden. Das AWCS könnte von einem unabhängigen Rat von Wirtschafts-, Sozial- und Umweltexpert:innen erstellt werden, die von EU-Parlament und EWSA berufen werden. Dieses Expertengremium könnte die relevanten Entwicklungen hinsichtlich Wohlstand, Konvergenz und Nachhaltigkeit qualitativ bewerten (Feigl, 2017).

Um Transparenz, Verantwortung und demokratische Teilhabe in EU-Entscheidungen zur Fiskalpolitik zu erhöhen und sicherzustellen, dass das AWCS hinreichende Aufmerksamkeit erhält, wäre es eine Möglichkeit, die von den einzelnen Minister:innen in der Eurogruppe vertretenen politischen Positionen öffentlich zu machen, damit eine qualifizierte Diskussion darüber auf der jeweils nationalen Ebene erfolgen kann. Eine zweite Möglichkeit bestünde darin, die Mitspracherechte des Europäischen Parlaments bezüglich der Finanzpolitik zu erweitern, sodass die europäischen Bürger:innen tatsächlich für oder gegen bestimmte Vorschläge und Positionen ihre Stimme abgeben könnten. Längerfristig könnte dafür auch ein eigenes Parlament für den Euroraum geschaffen werden, etwa als Untergruppe des EU-Parlaments, mit Mitbestimmungsrechten bei allen den Euroraum betreffenden wirtschaftspolitischen Entscheidungen (Andor et al., 2018).

Nach der erfolgreichen Verabschiedung des EU-Wiederaufbaufonds sollte sich die EU nun dringend an Reformen des fiskalischen Regelwerks begeben. Die begonnene Überprüfung der ökonomischen Governance sollte sie dabei als Chance für den Einstieg in eine mutige und weitreichende Reform nutzen.



Literatur

Álvarez, N., G. Feigl, N. Koratzanis, M. Marterbauer, M. Mathieu, T. McDonnell, L. Pennacchi, C. Pierros, H. Sterdyniak, A. Truger und J. Uxó (2019), Towards a Progressive EMU Economic Governance, ETUI Working Paper, 2019.13, European Trade Union Institute.
Andor, L. et al. (2018), Blueprint for a Democratic Renewal of the Eurozone, https://www.politico.eu/article/opinion-blueprint-for-a-democratic-renewal-of-the-eurozone/ (2. Februar 2021).
Bom P Ligthart J What Have We Learned From Three Decades of Research on the Productivity of Public Capital? Journal of Economic Surveys 2014 28 5 889 916 10.1111/joes.12037
Dullien, S., A. Herzog-Stein, K. Rietzler, S. Tober und S. Watzka (2021), Wirtschaftspolitische Herausforderungen 2021: Die Erholung nachhaltig gestalten, IMK Report, 164, Institut für Makroökonomie und Konjunkturforschung der Hans-Böckler-Stiftung.
European Fiscal Board (2020), Annual Report 2020.
EU-Kommission (2020a): Annual Macro-Economic Database (Ameco), Autumn.
EU-Kommission (2020b), Recovery and Resilience Facility — Grants allocation per Member State, https://ec.europa.eu/info/sites/info/files/about_the_european_commission/eu_budget/recovery_and_resilience_facility_.pdf (2. Februar 2021).
Europäischer Rat (2015), Commonly Agreed Position on Flexibility in the Stability and Growth Pact, 14345/15 ECOFIN 888 UEM 422.
Feigl, G. (2017), From growth to well-being: a new paradigm for EU economic governance, Policy Brief, 2/2017, European Trade Union Institute.
Gechert S What fiscal policy is most effective? A meta-regression analysis Oxford Economic Paper 2015 67 3 553 580 10.1093/oep/gpv027
Mersch, M. (Hrsg.) (2018), Well-being for everyone in a sustainable Europe. Report of the independent commission for sustainable equality, https://www.progressivesociety.eu/publication/report-independent-commission-sustainable-equality-2019-2024 (2. Januar 2021).
Seikel D Truger A Die blockierte Vollendung der Europäischen Währungsunion: Plädoyer für eine pragmatische Nutzung von fiskalischen Handlungsspielräumen Wirtschaft und Gesellschaft 2019 45 1 43 65
Stiglitz, J. E., J.-P. Fitoussi und M. Durand (2018), Beyond GDP. Measuring what counts for economic and social performance, OECD Publishing.
SVR [Sachverständigenrat zur Begutachtung der gesamtwirtschaftlichen Entwicklung] (2020), Corona-Krise gemeinsam bewältigen, Resilienz und Wachstum stärken, Jahresgutachten 2020/21.
Truger A The Fiscal Compact, Cyclical Adjustment and the Remaining Leeway for Expansionary Fiscal Policies in the Euro Area Panoeconomicus 2015 62 2 157 175 10.2298/PAN1502157T
Truger, A. (2015b), Implementing the Golden Rule for Public Investment in Europe- Safeguarding Public Investment and Supporting the Recovery, Materialien zu Wirtschaft und Gesellschaft, 138.
Truger A Reforming EU Fiscal Rules: More Leeway, Investment Orientation and Democratic Coordination Intereconomics 2020 55 5 277 281 10.1007/s10272-020-0915-z
Truger, A. (2021), Europa nach Corona: Für eine progressive Reform der Fiskalregeln, in A. Gärber (Hrsg.): Europa. Besser. Machen, erscheint demnächst.
UN (2015), Transforming our world: the 2030 agenda for sustainable development. Resolution adopted by the general assembly on 25 September 2015, https://sustainabledevelopment.un.org/post2015/transformingourworld (2. Februar 2021).
